# A Century of Mathematical Epidemiology: A Bibliometric Analysis and Visualization of Research Trends

**DOI:** 10.7759/cureus.71001

**Published:** 2024-10-07

**Authors:** Pratheesh Mathew, Dary John, Jais Kurian, Tony Cherian, Jobin Jose

**Affiliations:** 1 Mathematics, Nirmala College, Muvattupuzha, IND; 2 Mathematics, Newman College, Thodupuzha, IND; 3 Mathematics, St. Stephen's College, Uzhavoor, IND; 4 Library, Peet Memorial Training College, Mavelikara, IND; 5 Library, Marian College Kuttikkanam, Kuttikkanam, IND

**Keywords:** bibliometric analysis, biblioshiny, epidemiology, mathematical epidemiology, mathematics, vosviewer

## Abstract

Defined as the application of mathematical models and methods for the study of disease spread and control, Mathematical epidemiology has now emerged as a very important area for understanding public health dynamics. The paper presents an overall bibliometric analysis of research in mathematical epidemiology using the Scopus database. This overview comprises 1,787 documents: journal articles, book chapters, and conference papers from 819 sources. From 1916 to 2024, it has been possible to identify key trends, influential authors, and central themes through the application of the PRISMA methodology. The results reflect that since 2000, there has been a significant growth in research production; most of it was during the period of the COVID-19 pandemic. The study also determined trends in international collaboration, leading funding sponsors, and the dynamics underlying major research topics. According to this study, the role of mathematical models in epidemiology is becoming increasingly prominent, driven by the need to address complex global health challenges and an expanding influence on public health strategies.

## Introduction and background

The discipline of mathematical epidemiology has recently emerged as one in which mathematical modeling is combined with epidemiological inquiry to understand and predict infectious diseases. In that capacity, the role of mathematical models, especially the SIR (Susceptible-Infected-Recovered) model, has been instrumental in helping humankind understand the dynamics of infectious diseases. Over the past decade, there has been enormous success in applying such models to real-world scenarios, in part due to developments related to complex networks and computational tools that make more accurate modeling of disease spread and intervention effectiveness possible [1]. Bayesian approaches are also applied with increasing frequency to sharpen epidemiological models on data from high-resolution networks. It is in this light that such models are needed for enhanced estimates of parameters and assessments of risks related to public health scenarios, so their utility in the modeling of Shiga toxin-producing Escherichia coli shall be clearly elaborated in Sweden [2].

Computational approaches have become part and parcel of mathematical epidemiology these days, aided by the tools available from big data and artificial intelligence. The capacities most commonly used in a number of simulations that study spreading dynamics related to viral epidemics, such as ebola and influenza, have been strengthened for modeling disease dynamics in more realistic ways [3]. Recent studies have pointed out that psychological models should be an intrinsic part of the mathematical framework in order to understand how, given a disease, human behavior can lead to its spread. This interdisciplinarity is important in work striving to improve the accuracy of models so that there could be useful public health strategies [4].

Despite these advances, mathematical models are often challenged by the interpretation of noisy epidemiological data. New approaches have been conceived to distinguish true parametric heterogeneity from random noise to allow for more robustness and precision in making predictions of an epidemic [5]. New methodologies, like the one by the SBDiEM spatiotemporal model, have been introduced to capture intricacies in infectious disease dynamics. These models are specifically set out to integrate different scales of interaction, from micro-pathogens to macro-scale environmental factors, boosting the robustness of outbreak predictions [6]. Artificial intelligence and neural networks, in particular, have been used for the optimization of the coupling of complex models with real data. This approach allows for more efficient and accurate projections for diseases, as in the case of the COVID-19 modeling within Germany [7].

The intrinsic unpredictability of an epidemic process, driven by human behavior and environmental conditions, makes the task of modeling a bit difficult. However, lately, thermodynamic theories have been put forward to underpin a more holistic understanding of the dynamics of epidemics [8]. In such a case, mathematical models have been quite indispensable in view of the limitations of data during epidemics. A mathematical model, under rigorous statistical methods, can estimate a key epidemiological parameter even in cases where direct measurement is unavailable [9]. The future of mathematical epidemiology lies in further integration of interdisciplinary approaches with enhancements of computational tools and improving existing models to be more precise in tackling the complexities of disease dynamics [10].

Mathematical epidemiology has a deep historical foundation, evolving from early models designed to describe simple epidemic processes to more advanced frameworks capable of addressing the complexities of modern infectious disease dynamics. The field began with early models that focused on basic disease transmission patterns, but over time it has expanded to incorporate more detailed aspects, such as human behavior, population variability, and environmental influences. These developments have made mathematical models invaluable for public health research, allowing for more accurate predictions of disease spread and the evaluation of various intervention strategies. With the integration of computational methods, including agent-based and network-based simulations, the field has gained further strength in exploring the outcomes of different control measures, offering critical insights for policy decisions. Mathematical epidemiology has significantly advanced in the past decade, especially by combining computational with interdisciplinary approaches. These models will, therefore, become of key importance in managing public health and mitigating the impact of infectious diseases as the field continues to evolve.

The bibliometric analysis, having become indispensable across a wide range of disciplines, would be considered a very strong quantitative tool in the assessment and visualization of academic literature trends [11-15]. This technique identifies patterns and trends by undertaking systematic reviews of publications, citations, and other scholarly outputs, and it allows researchers to identify pivotal works and map the intellectual structure underlying a particular field of study [16-18]. By revealing these patterns, bibliometric analysis not only facilitates the identification of key research areas and influential authors but also aids in understanding the evolution of academic discourse over time [19].

Biblioshiny is a web-based, user-friendly interface for the R-based bibliometric package that allows researchers to easily run a comprehensive analysis with visualizations that do not require much programming [20,21]. The tool shall facilitate researchers to easily undertake the most complex tasks regarding bibliometric data and find meaningful insights from the same [22-25]. Similarly, VOSviewer has grown to become one of the most used tools for the generation and analysis of bibliometric networks, particularly in the domains of co-authorship, co-citation, and keyword co-occurrence [26,27]. Since VOSviewer is very strong on the visualization side, it has turned out to be a very helpful tool for researchers who need to learn about complex research landscapes through a clear presentation of relationships and clusters of large datasets [28-30]. The combination of these tools together allows for in-depth exploration into intellectual trends and dynamics from different domains.

The general purpose of the study is to conduct a proper bibliometric analysis with respect to the field of mathematical epidemiology, considering the evolution of key research trends, contributors, and sources as they have changed through time. Specifically, this study seeks to identify and analyze bibliometric measures for publications from 1916 to 2024, assess their impact and collaboration patterns among the authors, and describe the thematic development within the field. It also aims to draw a mapping of the co-authorship networks across countries, investigate the prevalence and co-occurrence of keywords, and underline the contribution of the different funding sponsors. These analyses will be able to give a detailed description of the research landscape regarding mathematical epidemiology.

## Review

Methodological approach

**Figure 1:**
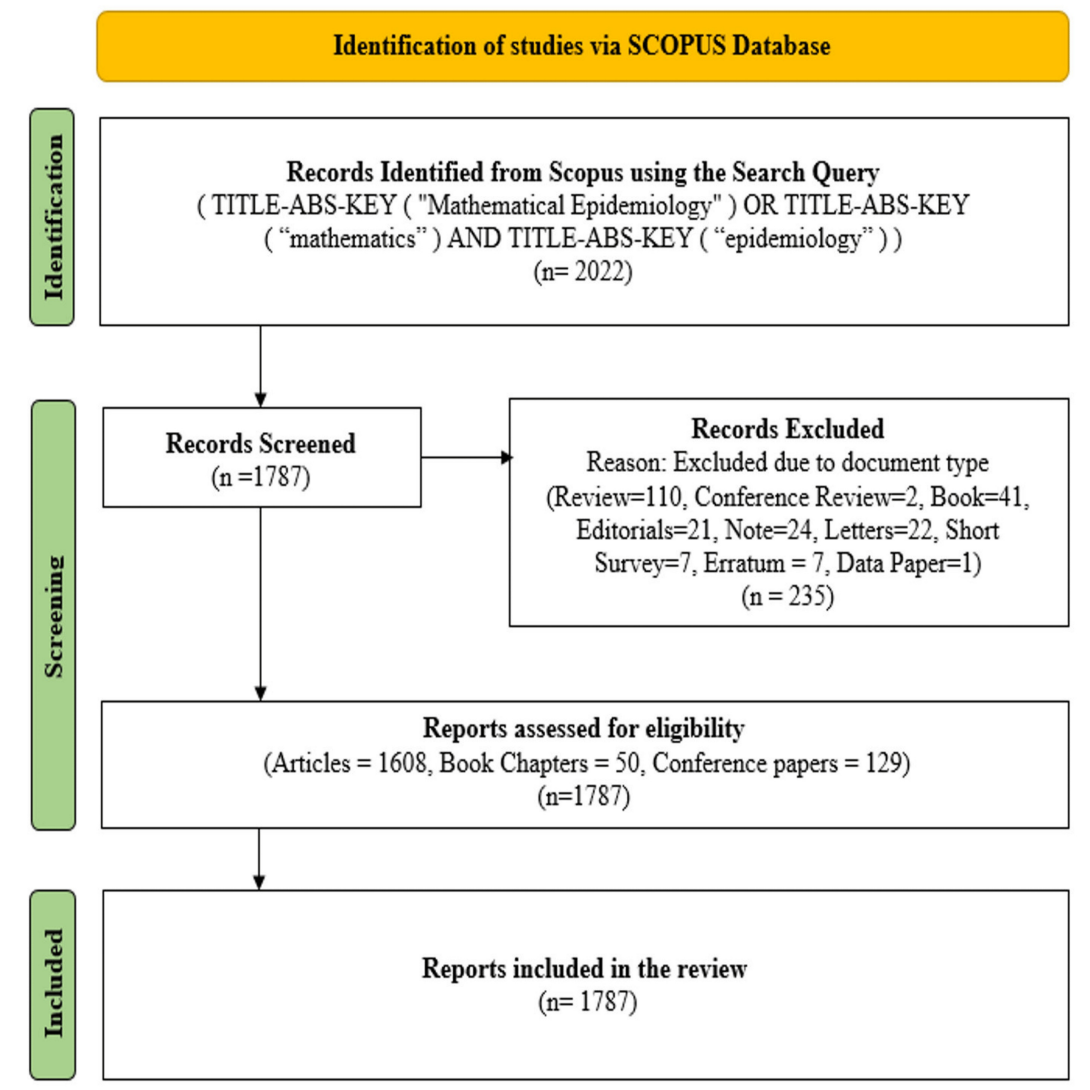
PRISMA flowchart

In this study, Scopus was selected as the principal source of bibliographic data due to its extensive collection of high-quality journals, which provides broader coverage compared to other databases. The publications were identified using the keywords "Mathematical Epidemiology," OR "Mathematics," AND "Epidemiology," without imposing language restrictions, with a focus on journal articles, book chapters, and conference papers. This search process resulted in the retrieval of 1,787 documents from 819 distinct sources, covering the period from 1916 to 2024. Figure 1 presents the PRISMA methodology adopted for paper selection in the bibliometric analysis, which followed a structured three-step process. Initially, relevant data were identified and extracted from the databases. The second step involved excluding document types such as reviews, editorials, letters, notes, and short surveys, thereby refining the selection to articles, book chapters, and conference papers. The extracted data were then saved in CSV format, and the subsequent bibliometric analysis was performed using VOSviewer and Biblioshiny software.

Table 1 provides a comprehensive overview of the dataset, highlighting its breadth and depth across various dimensions. The data spans over a century, from 1916 to 2024, encompassing 1,787 documents sourced from 819 journals, books, and other sources. With an annual growth rate of 3.97%, the dataset exhibits a consistent expansion in the field. The documents have an average age of 20 years and garner an impressive average of 47.6 citations per document, underscoring their scholarly impact. The dataset is well-cited, with a total of 47,077 references. The keyword analysis reveals a rich diversity of topics, with 8,042 keywords plus (ID) and 3,424 author's keywords (DE). The author collaboration data indicates a highly collaborative research environment, with 4,646 contributing authors, of whom 348 have single-authored documents. Notably, 21.49% of the works involve international co-authorship, reflecting a significant level of global collaboration. The document types are predominantly articles (1,608), with a smaller representation of book chapters (50) and conference papers (129), indicating a focus on peer-reviewed journal publications. This summary showcases the dataset's extensive coverage and the collaborative nature of research in this domain.

**Table 1:** Key findings of the investigation

Description	Results
Main information about the data	
Timespan	1916:2024
Sources (journals, books, etc.)	819
Documents	1787
Annual growth rate %	3.97
Document average age	20
Average citations per doc	47.6
References	47077
Document contents	
Keywords plus (ID)	8042
Author's keywords (DE)	3424
Authors	
Authors	4646
Authors of single-authored docs	348
Authors collaboration	
Single-authored docs	379
Co-authors per doc	3.13
International co-authorships %	21.49
Document types	
Article	1608
Book chapter	50
Conference paper	129

Trends in research publications

**Figure 2:**
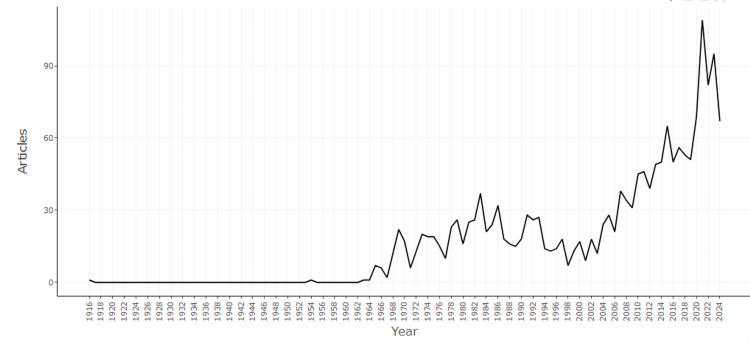
Annual scientific production

Figure 2 depicts the annual scientific production in the field of mathematical epidemiology from 1916 to 2024. The graph reveals a steady increase in the number of articles over time, with significant growth observed particularly after the year 2000. The trend shows a marked acceleration in publication activity starting around 2010, culminating in a peak between 2020 and 2022. This surge in research output could be attributed to global events, such as the COVID-19 pandemic, which likely spurred increased interest and investment in epidemiological studies. However, the slight decline after 2022 may indicate a stabilization or shifting focus in the research landscape. Overall, the data underscores the evolving and expanding interest in the intersection of mathematics and epidemiology, reflecting its growing importance in addressing public health challenges.

Key influential authors

Table 2 lists the most relevant authors in the field of mathematical epidemiology based on the number of articles they have contributed. Both Anderson RM and Wang W have made significant contributions with 10 articles each. They are followed closely by Chattopadhyay J, Greenhalgh D, Gumel AB, JR, Li J, and Li X, each with nine articles. Buonomo B and Feinstein AR have also made substantial contributions, each with eight articles. These authors are recognized for their pivotal roles in advancing research in this field, as reflected by their substantial publication records.

**Table 2:** Most relevant authors

Authors	Articles
Anderson Rm	10
Wang W	10
Chattopadhyay J	9
Greenhalgh D	9
Gumel AB	9
JR	9
Li J	9
Li X	9
Buonomo B	8
Feinstein AR	8

Authors' production over time

**Figure 3:**
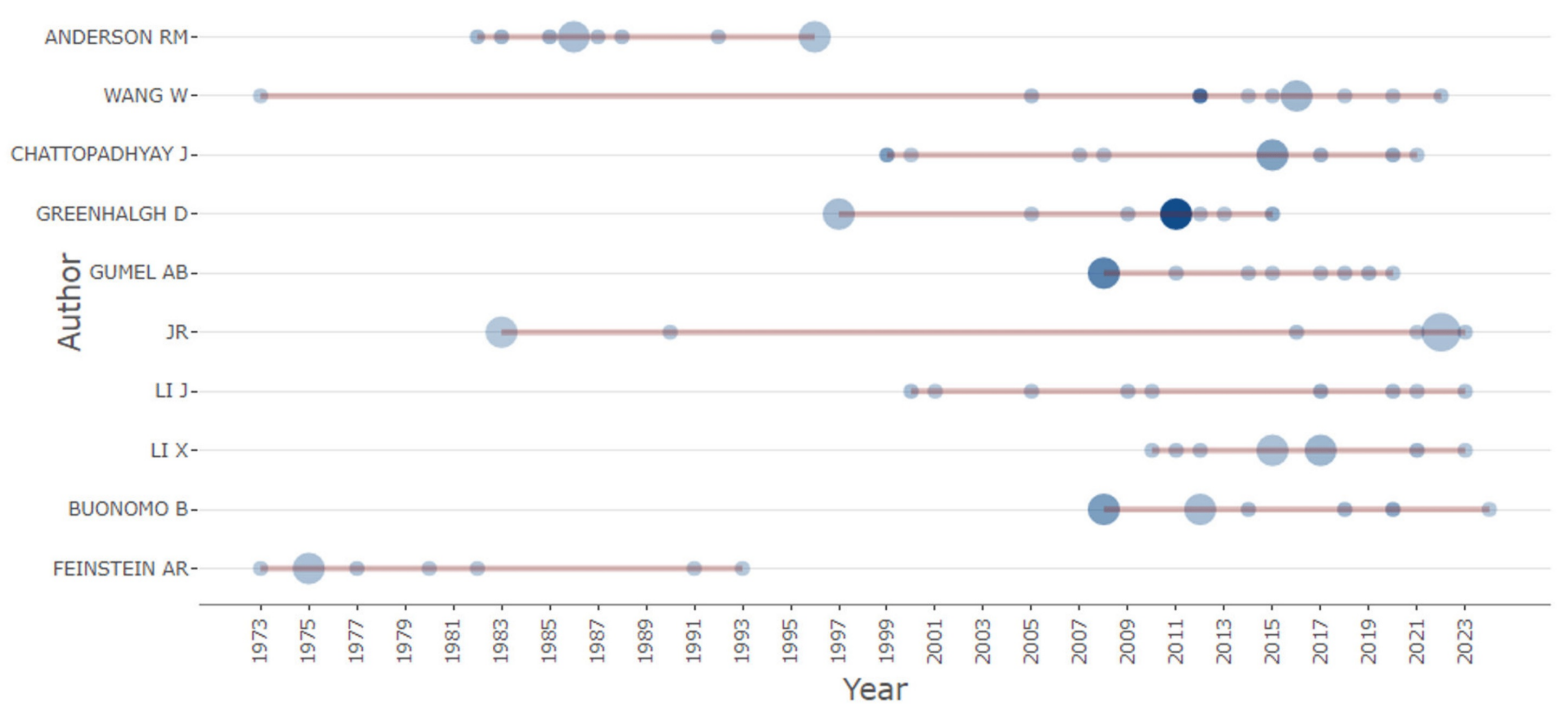
Authors' production over time The color of the circles represents the intensity of the research activity. Darker shades indicate higher levels of publication activity in a particular year. The size of the circles corresponds to the number of publications by the author in a given year. Larger circles indicate a higher number of publications.

Figure 3 illustrates the production of the most relevant authors in the field of mathematical epidemiology over time. The chart visualizes each author's publication activity, indicating both the number of articles published and the total citations per year (TC per year). Authors such as Anderson RM and Wang W, who are among the top contributors with 10 articles each, have demonstrated consistent research output over several decades. The size of the circles represents the volume of articles published, while the intensity of the color indicates the citation impact per year. This visualization highlights not only the sustained contributions of key authors but also the periods during which their work garnered significant academic attention, reflecting their influence in the field. Notably, some authors, like Greenhalgh D and Gumel AB, show peaks in their production and citation impact during specific years, suggesting pivotal contributions during those periods. Overall, the figure provides insight into the temporal distribution and impact of research by leading scholars in mathematical epidemiology.

Most relevant sources

Table 3 lists the most relevant sources in the field of mathematical epidemiology, ranked by the number of articles they have published. Mathematical Biosciences leads with 65 articles, indicating its prominent role in disseminating research in this domain. Following closely, the Journal of Mathematical Biology has contributed 46 articles, and Chaos, Solitons and Fractals has published 36 articles, reflecting their significant contributions to the field. Mathematical Biosciences and Engineering and the International Journal of Epidemiology have also made notable contributions, with 32 and 24 articles, respectively. Other important sources include the Journal of Microbiology, Epidemiology, and Immunobiology (23 articles), the Bulletin of Mathematical Biology (22 articles), and the American Journal of Epidemiology (21 articles). Mathematical Methods in the Applied Sciences and Plos One round out the list, each with 21 and 19 articles, respectively. These journals represent the key platforms for publishing and advancing research in mathematical epidemiology.

**Table 3:** Most relevant sources

Sources	No. of articles
Mathematical Biosciences	65
Journal of Mathematical Biology	46
Chaos, Solitons and Fractals	36
Mathematical Biosciences and Engineering	32
International Journal of Epidemiology	24
Journal of Microbiology, Epidemiology and Immunobiology	23
Bulletin of Mathematical Biology	22
American Journal of Epidemiology	21
Mathematical Methods in the Applied Sciences	21
Plos One	19

Trending Topics

**Figure 4:**
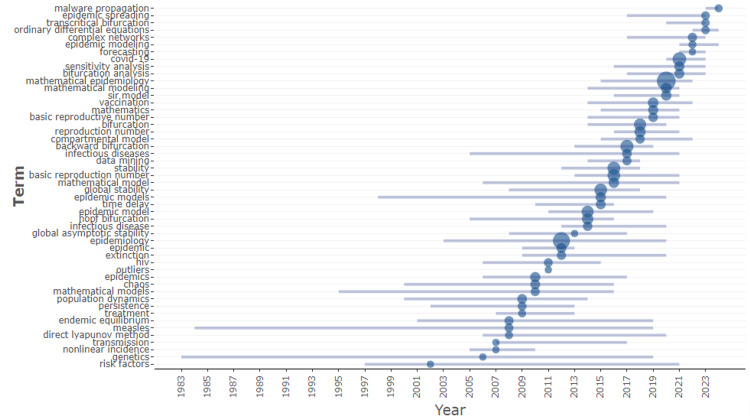
Trending topics

Figure 4 represents the timeline of trending topics in mathematical epidemiology visualizing how terms have surged to prominence through time in the academic literature. For a key term, Figure 4 represents the trend in mathematical epidemiology from the early 1970s until 2024. Term frequency is denoted by the size of circles, where a larger circle stands for a higher frequency of term occurrence. The main relative increases are for terms such as "basic reproduction number," "epidemic model," and "mathematical model," especially over the years between 2000 and later, thus demonstrating increased research interest. It also shows the emergence of more specialized topics in recent years prolifically, such as "global asymptotic stability," "endemic equilibrium," and "nonlinear dynamics." This trend captures the evolution of the field, particularly in relation to advancements in the ability to perform simulations using mathematical models and the global impact of infectious diseases like COVID-19. As a result, researchers have increasingly focused on more complex and specific aspects of epidemiology. In this way, the visualization captures the dynamics of research topics in mathematical epidemiology and the way the scholarly focus has changed or expanded over time.

Thematic map

**Figure 5:**
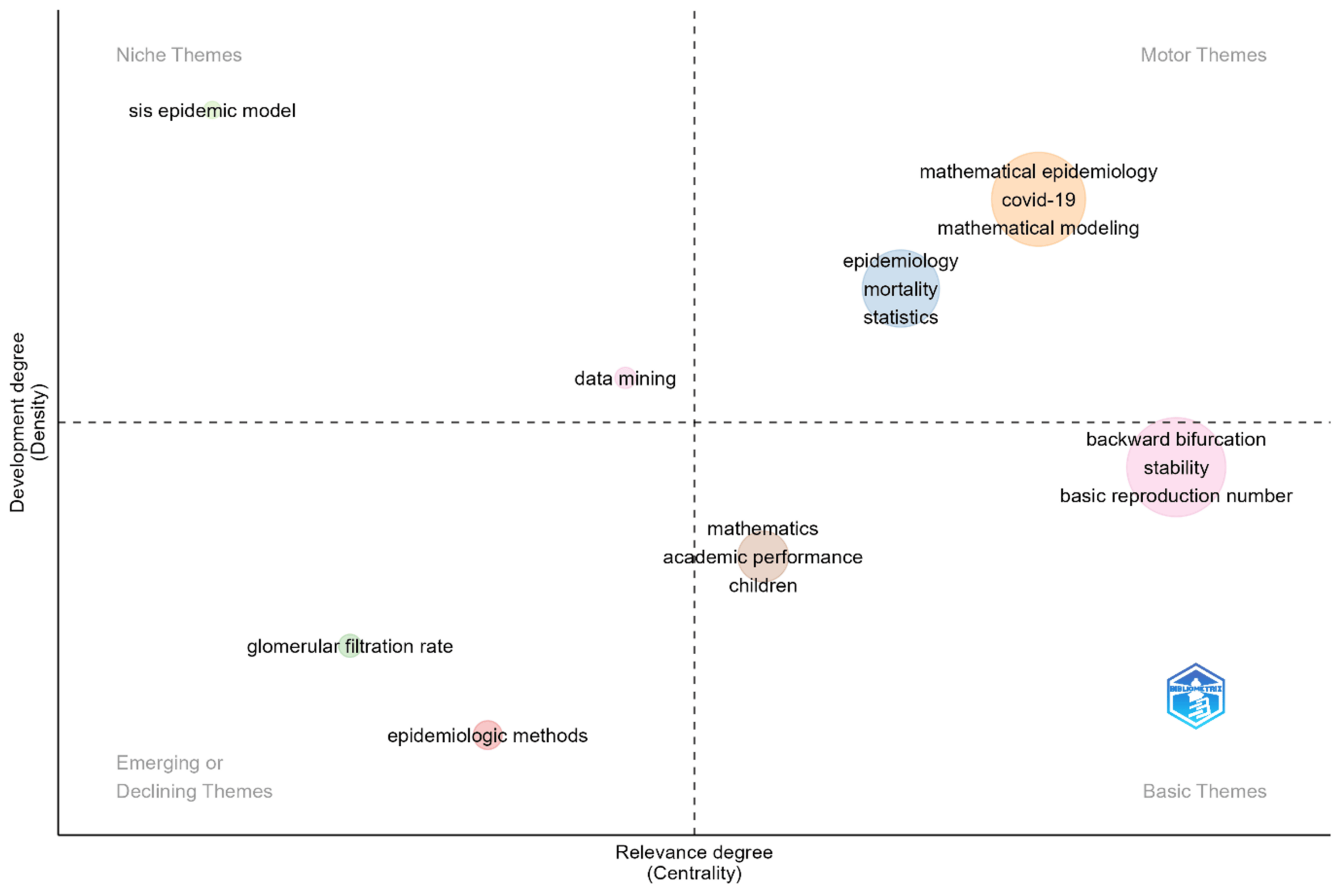
Thematic map

Figure 5 presents a thematic map where research themes in mathematical epidemiology are ordered by degree of relevance expressed as centrality and level of development expressed as density. It is divided into four quadrants separating different kinds of themes. This is a result of the well-developed and highly relevant themes; motor themes in the upper right quadrant include "mathematical epidemiology," "COVID-19," and "mathematical modeling." Themes like these occupy the middle ground of importance for driving against important research activity. Developed themes that are highly specialized find a place in the niche themes quadrant at the higher, left part of the map. An example of this might include the "SIS epidemic model." The basic themes quadrant in the bottom right are high in relevance but low in development density, such as basic reproduction number, stability, and backward bifurcation, which thus constitute the skeleton of an underlying theoretical framework or give substance to the field in question. The themes identified with the emerging or declining quadrants are the ones at the low left, such as "epidemiologic methods" and "glomerular filtration rate," which are either at a very nascent stage of development or have lost relevance. These provide a comprehensive view of the current research landscape in mathematical epidemiology, highlighting important areas where research may be focused.

Three-field plot

**Figure 6:**
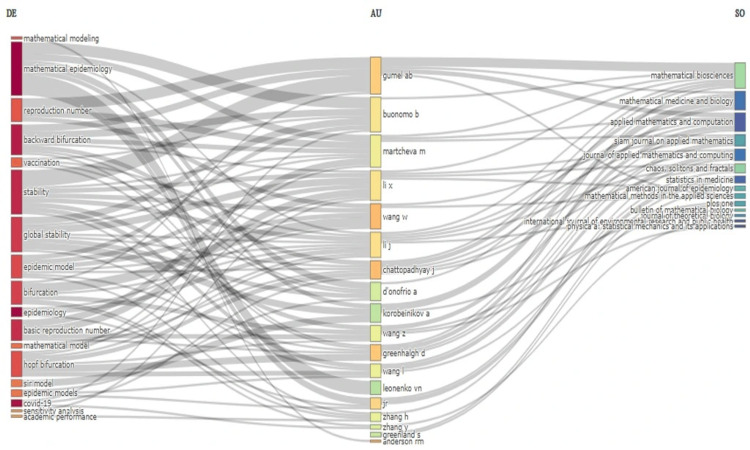
Three-field plot showing the connections between keywords, authors, and publication sources

Figure 6 displays a three-field plot that illustrates the connections between keywords (DE), authors (AU), and publication sources (SO) within the field of mathematical epidemiology. The left-hand side of the plot allows locating the relevance of significant key terms being prevalent in the literature such as "Mathematical Modeling," Mathematical Epidemiology," and "Reproduction Number." These keywords are linked to the central field of authors, pointing out the researchers with significant contributions to this area, such as Gumel AB, Buonomo B, and Martcheva M, among others. There are strong implications in the map for authors like Gumel AB, Buonomo B, and Martcheva M, which are typical of their common relevance and diversity in research work. This is summarized in the right-hand side linking to the publication sources Mathematical Biosciences and Mathematical Medicine and Biology, Applied Mathematics, and Computation. Clearly, with this figure, the multidisciplinary character of publications in Scientific Epidemiology is well manifested, along with the uplinking terms from the authors to where they publish. Also, the plot reveals the relatedness of the research ecosystem in the sense that some authors and journals have acted as the network's hubs in the dissemination of knowledge in this field.

Documents by funding sponsor

**Figure 7:**
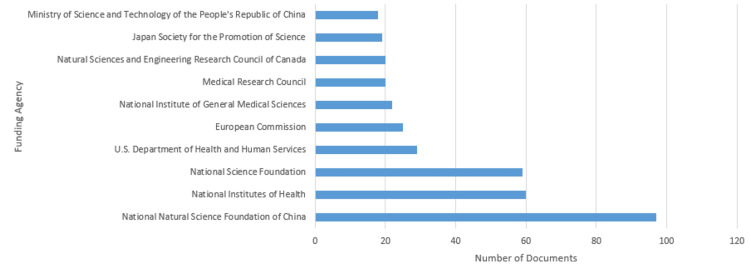
Documents by funding sponsor generated from SCOPUS

Figure 7 shows the document analysis sponsored according to the funding sponsor, pointing out the great contribution of several national or international organizations in support of mathematical epidemiology research. The National Natural Science Foundation of China, with 97 documents at the top, clearly demonstrates its strong commitment to advancing research in this field. These are followed by the National Institutes of Health with 60 funded documents and are closely tailed by the National Science Foundation with 59 documents. Backing for 29 of these documents has come from the U.S. Department of Health and Human Services, while 25 have been funded by the European Commission, indicating that interest in, and commitment to, epidemiological research is global in scope. Other major sponsors include the National Institute of General Medical Sciences with 22 documents, the Medical Research Council and the Natural Sciences and Engineering Research Council of Canada with 20 documents apiece, and the Japan Society for the Promotion of Science with 19. The Ministry of Science and Technology of the People's Republic of China rounds out this list with 18 funded documents. It underlines the very critical financial support these organizations have provided to the researchers in mathematical epidemiology for continued development and research dissemination.

Global collaborative networks

**Figure 8:**
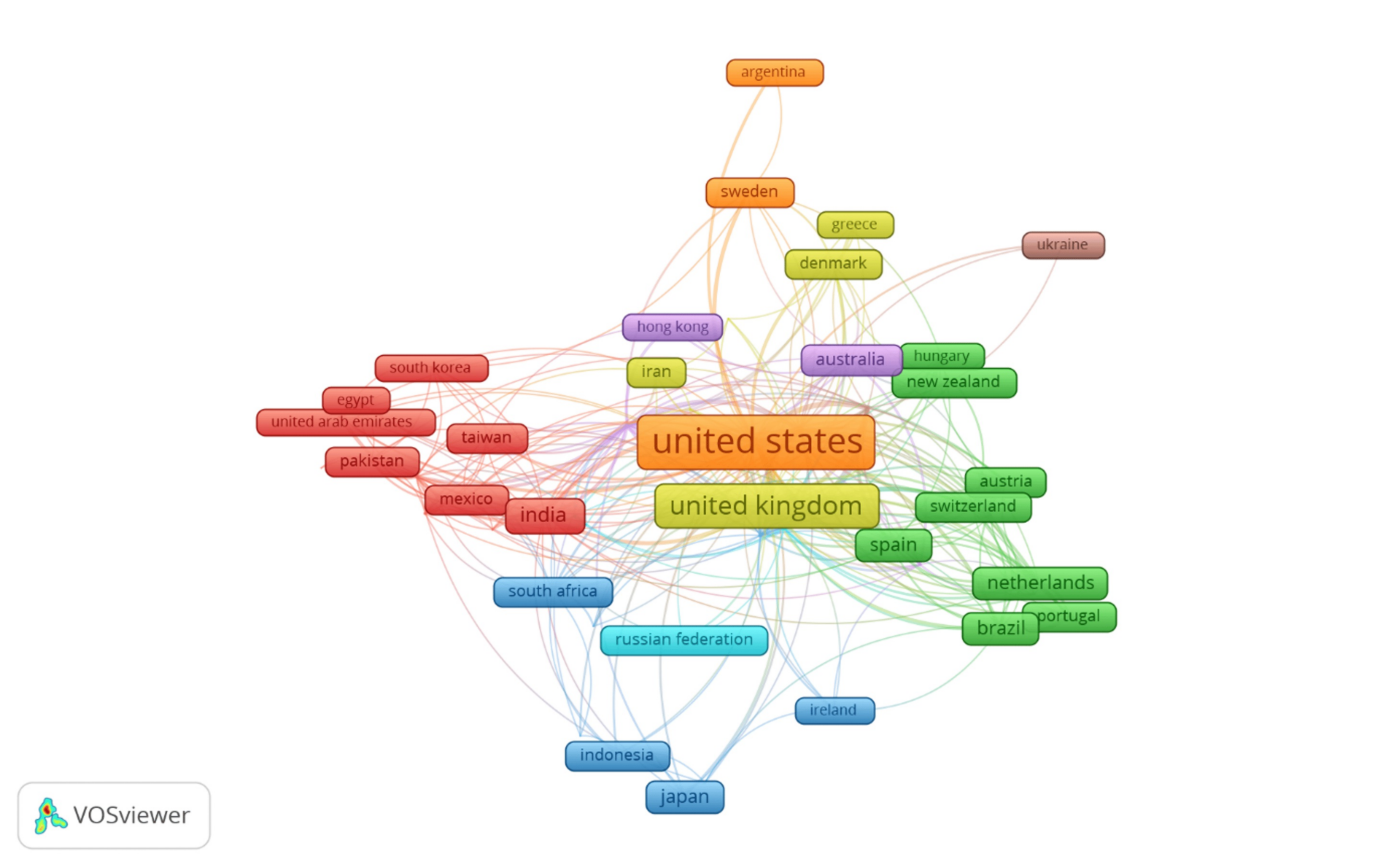
Co-authorship between countries

Figure 8 illustrates the co-authorship relationships between countries in the field of mathematical epidemiology. The network visualization shows the collaboration intensity among countries, with the size of each country’s label reflecting the extent of its contribution and the thickness of the connecting lines indicating the strength of the collaborative ties. The United States and the United Kingdom are prominently positioned at the center of the network, indicating their leading roles in international collaborations. These two countries exhibit strong co-authorship connections with many other nations, reflecting their influence and extensive collaboration in the field. Other countries such as China, India, Australia, and Canada also show significant collaboration networks, contributing to the global research output. Regions like Europe are well-represented with countries such as Spain, Switzerland, Netherlands, and Germany forming strong links both within the continent and with other parts of the world. Asia, Japan, South Korea, and China display robust connections, particularly with the United States, underscoring their collaborative efforts in epidemiological research. The map also highlights emerging collaborations involving countries like Brazil, South Africa, and Mexico, indicating their growing involvement in global research networks. The co-authorship network visualized in this figure underscores the interconnected nature of research in mathematical epidemiology, where international collaboration plays a pivotal role in advancing the field.

Co-occurrence of keywords

**Figure 9:**
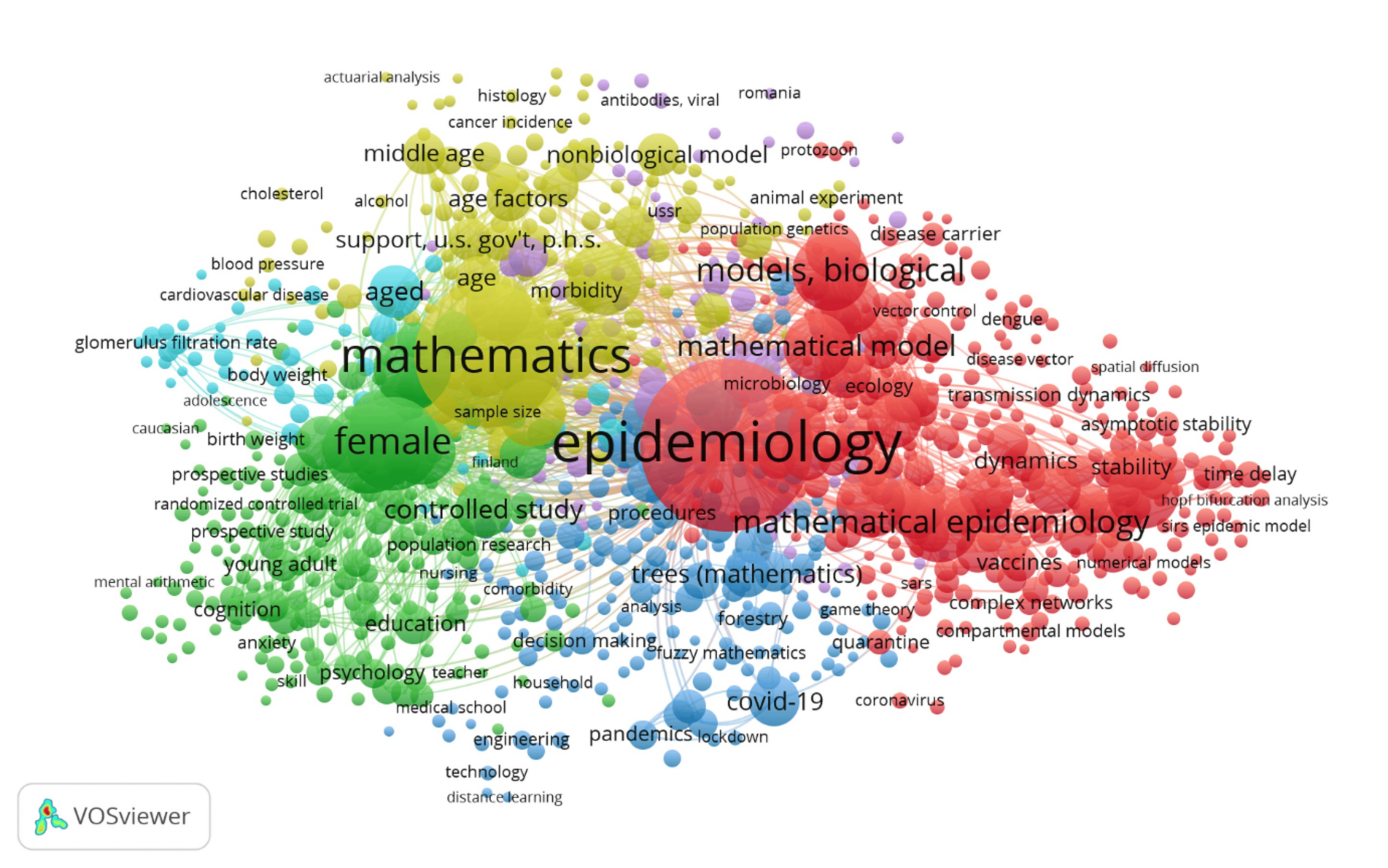
Co-occurrence of keywords

Figure 9 presents a visualization of the co-occurrence of keywords in the field of mathematical epidemiology. This map indicates how often different keywords co-occur in the literature. The size of a keyword reflects how often this term appears, and the proximity of two keywords determines their co-occurrence. Unsurprisingly, the two core keywords in this map are "Epidemiology" and "Mathematics," indicating a central positioning for both terms in the research domain. These terms would be very strongly connected to other key terminology such as "Mathematical Epidemiology," "Mathematical Model," and "Biological Models," which are very close together, underlining their interest in mathematical modeling during epidemiological research. The map displays the coded colors that turn out into clear-cut clusters; each represents a grouping of related keywords that often co-occur. For example, one cluster may be dealing with mathematical aspects of epidemiology, with terms like "Models, Biological," "Stability," and "Dynamics." Another may orient toward biological or health-related/relevant issues, with keywords like "Pandemics," "COVID-19," and "Infectious Diseases." This visualization effectively demonstrates the interconnectedness of various research themes within mathematical epidemiology, showcasing how different aspects of mathematics and epidemiology are integrated into the literature. It also highlights emerging and well-established research areas, providing insights into the current trends and focus areas in the field.

Discussion

The bibliometric analysis presented here describes the research landscape in the field of mathematical epidemiology authoritatively, covering more than a century from 1916 to 2024. The data shows an increase in scientific output for this specialty and further proves that scientific production has indeed grown stronger after major global health crises, such as COVID-19. This increase reached its peak between 2020 and 2022 and very clearly demonstrates how much mathematical models contributed to understanding and controlling infectious diseases. This underscores the maturing field of research at the interface of mathematics and epidemiology toward global health.

The identification of key contributing authors and journals also puts across the key players driving this research domain. The core journals identified are Mathematical Biosciences and the Journal of Mathematical Biology, while core contributing authors include Anderson RM and Wang W.

Thematic analysis: A shift toward more specific topics, complex modeling approaches, and observations on the maturity of the field. The extent of international collaboration is very high, with a tight focus on the integration of mathematical concepts and epidemiology.

Research gaps and future directions

The study brings to light considerable gaps and leaves room for further research. One of these gaps lies in the makeup of the research network because of underrepresentation from regions like Africa and Latin America. Further research into addressing this may, therefore, set up avenues for collaboration that may lead to the development of locally relevant epidemiological models. This also goes for the territories by which the mathematical epidemiology could head, including such aspects as the incorporation of new technologies like machine learning and artificial intelligence that would improve prediction modeling and enhance real-time epidemic forecasting, hence tailoring public health interventions.

There is also a need for more longitudinal studies that track the long-term impact of key models on public health policy and decision-making. These studies would go a long way in showing the impact foundational models have on future research and applications for disease management. Furthermore, the ethical implications of using mathematical models in public health deserve greater attention. Future work should center on transparency, fairness, and bias of algorithmic models with a focus on data privacy. This could also be applied to non-communicable diseases, such as cancer or diabetes, where mathematical modeling may be effectively used to probe new avenues of prevention and strategies of management within public health.

Practical implications

These findings bring out important practical implications for researchers, policymakers, and public health professionals. This surge in publications due to health crises, especially COVID-19, resulted in the expectation that mathematical models should be integrated into public health policymaking. The models will guide evidence-based decisions at the front end and guarantee efficiency in resource allocation during outbreaks. Third, there is an urgent call to integrate mathematical epidemiology into the curriculum. Consequently, mathematical modeling training programs will equip future researchers with the means to make their contribution to the growth of this field of research.

Another key practical consideration is that of funding for research. Organizations such as the National Natural Science Foundation of China and the National Institutes of Health have been instrumental in supporting study advances. Continued and increased funding related to supporting international collaborations and interdisciplinary research in the face of emerging global health challenges will be required. Such an approach will be increasingly required to share data, models, and methodologies in the strengthening of global research networks that will facilitate the development of real-time decision-support tools based on mathematical models. Tools from this collaboration would make public health systems across varied geographical regions more responsive by providing valuable insights into the prediction of outbreaks, resource management, and designs of interventions targeted at improvement in health outcomes.

## Conclusions

The bibliometric analysis in mathematical epidemiology reflects a dynamic field in evolution that has grown tremendously over the last century. Data shows a significant surge in research output, particularly in response to global events such as the COVID-19 pandemic, raising interest and investment in epidemiological studies. The analysis identifies key contributors and sources that have played a central role in the advancement of the field, with mathematical modeling being a crucial tool in understanding and addressing public health challenges. Notably, it is also cooperative research, with international co-authorship and funding support from big organizations across the world. The thematic analysis shows that the recent content, and above all the emerging topics, have been of a more specialized and complex nature. This provides a nuanced overview of the current status of mathematical epidemiology for researchers, policymakers, and practitioners interested in the intersection of mathematics and public health.

## References

[1] Mata AS, Dourado SM (2021). Mathematical modeling applied to epidemics: an overview. São Paulo J Math Sci.

[2] Engblom S, Eriksson R, Widgren S (2020). Bayesian epidemiological modeling over high-resolution network data. Epidemics.

[3] Shil P (2016). Mathematical modeling of viral epidemics: a review. Biomed Res J.

[4] Orr M, Mortveit HS, Lebiere C, Pirolli P (2023). A 10-year prospectus for mathematical epidemiology. Front Psychol.

[5] Romero-Severson EO, Ribeiro RM, Castro M (2018). Noise is not error: detecting parametric heterogeneity between epidemiologic time series. Front Microbiol.

[6] Bekiros S, Kouloumpou D (2020). SBDiEM: a new mathematical model of infectious disease dynamics. Chaos Solitons Fractals.

[7] Radev ST, Graw F, Chen S, Mutters NT, Eichel VM, Bärnighausen T, Köthe U (2021). OutbreakFlow: model-based Bayesian inference of disease outbreak dynamics with invertible neural networks and its application to the COVID-19 pandemics in Germany. PLoS Comput Biol.

[8] Koivu-Jolma M, Annila A (2018). Epidemic as a natural process. Math Biosci.

[9] Cauchemez S, Hoze N, Cousien A, Nikolay B, Ten Bosch Q (2019). How modelling can enhance the analysis of imperfect epidemic data. Trends Parasitol.

[10] Arino J (2020). Mathematical epidemiology in a data-rich world. Infect Dis Model.

[11] Kim MC, Chen C (2015). A scientometric review of emerging trends and new developments in recommendation systems. Scientometrics.

[12] Chen Y, Dong Y, Zeng Y (2020). Mapping of diseases from clinical medicine research—a visualization study. Scientometrics.

[13] Calof J, Søilen KS, Klavans R, Abdulkader B, Moudni IE (2022). Understanding the structure, characteristics, and future of collective intelligence using local and global bibliometric analyses. Technol Forecast Soc Change.

[14] Hajkowicz S, Sanderson C, Karimi S, Bratanova A, Naughtin C (2023). Artificial intelligence adoption in the physical sciences, natural sciences, life sciences, social sciences and the arts and humanities: A bibliometric analysis of research publications from 1960-2021. Technol Soc.

[15] do Carmo G, Felizardo LF, de Castro Alcântara V, da Silva CA, do Prado JW (2023). The impact of Jürgen Habermas's scientific production: a scientometric review. Scientometrics.

[16] Donthu N, Kumar S, Mukherjee D, Pandey N, Lim WM (2021). How to conduct a bibliometric analysis: an overview and guidelines. J Bus Res.

[17] Brika SKM, Algamdi A, Chergui K, Musa AA, Zouaghi R (2021). Quality of higher education: a bibliometric review study. Front Educ.

[18] Cruz-Cárdenas J, Zabelina E, Guadalupe-Lanas J, Palacio-Fierro A, Ramos-Galarza C (2021). COVID-19, consumer behavior, technology, and society: a literature review and bibliometric analysis. Technol Forecast Soc Change.

[19] Racine J (2012). RStudio: a platform-independent IDE for R and Sweave. J Appl Econometrics.

[20] Büyükkıdık S (2022). A bibliometric analysis: a tutorial for the bibliometrix package in R using IRT literature. J Meas Eval Educ Psychol.

[21] Thangavel P, Chandra B (2023). Two decades of M-commerce consumer research: a bibliometric analysis using R biblioshiny. Sustainability.

[22] Sidhu AK, Singh H, Virdi SS, Kumar R (2020). A bibliometric analysis on job stress using visualizing network. JCCC.

[23] Ejaz H, Zeeshan HM, Ahmad F (2022). Bibliometric analysis of publications on the Omicron variant from 2020 to 2022 in the Scopus database using R and VOSviewer. Int J Environ Res Public Health.

[24] Savita Savita, Verma N (2020). A review study on big data analysis using R Studio. Int J Eng Tech Mgmt Res.

[25] Thomas B, Joseph J, Jose J (2023). Explorative bibliometric study of medical image analysis: unveiling trends and advancements. SciVis.

[26] van Eck NJ, Waltman L (2010). Software survey: VOSviewer, a computer program for bibliometric mapping. Scientometrics.

[27] Agbo FJ, Oyelere SS, Suhonen J, Tukiainen M (2021). Scientific production and thematic breakthroughs in smart learning environments: a bibliometric analysis. Smart Learn Environ.

[28] Yu Y, Li Y, Zhang Z (2020). A bibliometric analysis using VOSviewer of publications on COVID-19. Ann Transl Med.

[29] Wong D (2018). VOSviewer. Tech Serv Q.

[30] Arruda H, Silva ER, Lessa M, Proença D Jr, Bartholo R (2022). VOSviewer and bibliometrix. J Med Libr Assoc.

